# A strategy for molecular diagnostics of Fanconi anemia in Brazilian patients

**DOI:** 10.1002/mgg3.293

**Published:** 2017-05-09

**Authors:** Daniela V. Pilonetto, Noemi F. Pereira, Carmem M. S. Bonfim, Lisandro L. Ribeiro, Marco A. Bitencourt, Lianne Kerkhoven, Karijn Floor, Najim Ameziane, Hans Joenje, Johan J. P. Gille, Ricardo Pasquini

**Affiliations:** ^1^ Immunogenetics Laboratory Hospital de Clínicas Universidade Federal do Paraná Curitiba PR Brazil; ^2^ Bone Marrow Transplantation Service Hospital de Clínicas Universidade Federal do Paraná Curitiba PR Brazil; ^3^ Department of Clinical Genetics VU University Medical Center Amsterdam The Netherlands

**Keywords:** *FANCA*, *FANCC*, *FANCG*, Fanconi anemia, genetic subtypes, molecular diagnostics, mutation screening

## Abstract

**Background:**

Fanconi anemia (FA) is a predominantly autosomal recessive disease with wide genetic heterogeneity resulting from mutations in several DNA repair pathway genes. To date, 21 genetic subtypes have been identified. We aimed to identify the FA genetic subtypes in the Brazilian population and to develop a strategy for molecular diagnosis applicable to routine clinical use.

**Methods:**

We screened 255 patients from Hospital de Clínicas, Universidade Federal do Paraná for 11 common FA gene mutations. Further analysis by multiplex ligation‐dependent probe amplification (MLPA) for *FANCA* and Sanger sequencing of all coding exons of *FANCA*,* ‐C*, and –*G* was performed in cases who harbored a single gene mutation.

**Results:**

We identified biallelic mutations in 128/255 patients (50.2%): 89, 11, and 28 carried *FANCA*,*FANCC*, and *FANCG* mutations, respectively. Of these, 71 harbored homozygous mutations, whereas 57 had compound heterozygous mutations. In 4/57 heterozygous patients, both mutations were identified by the initial screening, in 51/57 additional analyses was required for classification, and in 2/57 the second mutation remained unidentified. We found 52 different mutations of which 22 were novel.

**Conclusion:**

The proposed method allowed genetic subtyping of 126/255 (49.4%) patients at a significantly reduced time and cost, which makes molecular diagnosis of FA Brazilian patients feasible.

## Introduction

Fanconi anemia (FA) is a genome instability syndrome that affects multiple organs, and is characterized by a range of physical abnormalities, a predisposition to neoplasias, and stem cell loss leading to progressive bone marrow failure (Crossan and Patel 2012; Kee and D'Andrea 2012; Schneider et al. 2015).

Investigations regarding the molecular pathogenesis underlying FA have revealed a cellular mechanism that plays an important role in the maintenance of genome integrity. Upon the occurrence of DNA damage, upstream FA proteins (FANCA, ‐B, ‐C, ‐E, ‐F, ‐G, and ‐L) form a nuclear core complex. The FANCM complex (FANCM, FAAP24, FAAP16/MHF1, and FAAP10/MHF2) recruits the FA proteins of the core complex to the chromatin, where it is associated to FANCT/UBE2T forming the catalytic subunit E2/E3 that monoubiquitinates FANCD2 and FANCI. The FANCT/UBE2T provides the E2 ubiquitin‐conjugating activity and FANCL functions as the E3 ubiquitin ligase enzyme necessary for this process. The ubiquitinated FANCD2/FANCI heterodimer then interacts with downstream proteins (FANCD1/BRCA2, FANCJ/BRIP1, FANCN/PALB2, FANCO/RAD51C, FANCP/SLX4, FANCQ/XPF‐ERCC4, FANCR/RAD51, FANCS/BRCA1, FANCU/XRCC2, and FANCV/MAD2L2/REV7) and other nucleases such as FAN1, MUS81‐EME1, and SLX1 that mediate DNA damage response (Deans and West 2011; Kottemann and Smogorzewska 2013; Garaycoechea and Patel 2014; Bogliolo and Surrallés 2015; Dong et al. 2015; Wang and Smogorzewska 2015; Ceccaldi et al. 2016; Mamrak et al. 2016). Mutations in any of the 21 genes encoding these proteins disrupt this mechanism and may result in clinical and cellular characteristics observed in FA patients (De Winter and Joenje 2009; Vaz et al. 2010; Kim et al. 2011; Stoepker et al. 2011; Kee and D'Andrea 2012; Bogliolo et al. 2013; Ameziane et al. 2015; Hira et al. 2015; Rickman et al. 2015; Sawyer et al. 2015; Virts et al. 2015; Wang et al. 2015; Bluteau et al. 2016; Park et al. 2016).

FA can be caused by biallelic mutations in the majority of the genes, hemizygous mutations in *FANCB*, or by dominant negative mutations in *RAD51*. Over the last 2 years, five new FA genes were discovered, such as *FANCR/RAD51*,* FANCS/BRCA1*,* FANCT/UBE2T*,* FANCU/XRCC2*, and *FANCV/MAD2L2/REV7*. The rapid advancement in identification of new FA genes improves the understanding of the role of FA proteins in the DNA repair and has important implications in the molecular characterization of FA patients. (Vaz et al. 2010; Ameziane et al. 2015; Hira et al. 2015; Sawyer et al. 2015; Wang et al. 2015; Bluteau et al. 2016; Mamrak et al. 2016; Park et al. 2016).

The high phenotypic variability in patients with FA and the overlap of symptoms with other syndromes make the diagnosis difficult on the basis of clinical manifestations alone, and confirmation of clinical findings by laboratory methods becomes necessary. The classic FA diagnostic test is the detection of cellular hypersensitivity to DNA interstrand crosslinking agents such as diepoxybutane (DEB) and mitomycin C (Oostra et al. 2012; Auerbach 2015). However, a rapid and accurate diagnosis of FA is of great importance, as it would significantly affect patient follow‐up and treatment decisions. These requirements led to the development of diagnostic techniques based on molecular analysis (Castella et al. 2011; Ameziane et al. 2012; Gille et al. 2012; Aslan et al. 2015).

FA molecular diagnosis has been demanding and onerous due to the genetic heterogeneity associated with the disease. In addition to the number of genes involved, hundreds of unique causative mutations have been reported throughout the FA genes (Wijker et al. 1999; Gille et al. 2012; De Rocco et al. 2014). In various populations, founder mutations have been identified. Information on the ethnic background of the patient might provide evidence for a pathogenic mutation that is likely to be causal (Faivre et al. 2000; Tipping et al. 2001; Kutler and Auerbach 2004; Castella et al. 2011).

This study proposes a strategy for the molecular investigation of Brazilian FA patients based on an initial screening for common mutations in the most frequently affected genes *FANCA* (OMIM 607139), *FANCC* (OMIM 613899), and *FANCG* (OMIM 602956). In patients where both mutations are not identified by the initial screening approach, further investigation is performed using multiplex ligation‐dependent probe amplification (MLPA) and Sanger sequencing of the entire coding region of the genes. The molecular characterization of patients with FA is of major importance because it permits the exclusion of diseases with overlapping clinical symptoms, allows families to receive accurate genetic counseling, and facilitates the development of targeted prenatal genetic testing. In addition, accurate molecular stratification of patients is essential for participation in forthcoming gene therapy trials (Ameziane et al. 2008; Gille et al. 2012; Knies et al. 2012).

## Materials and Methods

### Ethical compliance

This study was approved by the HC/UFPR Ethical Committee on Human Research, and informed consent was obtained from subjects or their legal guardians.

### Patients

Our cohort included 255 Brazilian probands with FA diagnoses confirmed by chromosomal breakage (DEB) test (Auerbach 2015). Patients were followed at the Fanconi Anemia Outpatient Clinic ‐ Hospital de Clínicas, Universidade Federal do Paraná (HC/UFPR), between 1995 and 2012. All 255 patients were investigated by the proposed screening test, and the investigation proceeded with 128/255 patients in whom at least one FA mutation was identified.

### DNA extraction

Genomic DNA was isolated from peripheral blood samples according to Miller et al. (1988) using a modified salting out procedure.

### Strategy for molecular investigation of Brazilian patients with FA

Patients were initially screened for common mutations in the *FANCA*,* FANCC*, and *FANCG* genes. MLPA was used to detect *FANCA* large deletions, and Sanger sequencing of these genes was utilized when the second mutation was not identified either by common mutation screening or by MLPA. Both MLPA and Sanger sequencing methods were performed at the Department of Clinical Genetics, VU University Medical Center, Amsterdam, the Netherlands as part of a training that allowed the implementation of these methodologies to the Laboratory of Immunogenetics of HC/UFPR in Brazil.

### Screening of FA common mutations

The 11 commonly occurring mutations in the *FANCA*,* FANCC*, and *FANCG* genes were selected to comprise the initial screening panel (Table 1). The methods used to identify each of these mutations were polymerase chain reaction (PCR), amplification‐refractory mutation system PCR (ARMS‐PCR), and PCR‐restriction fragment length polymorphism (RFLP) as shown in Tables 2 and 3.

**Table 1:** Mutation screening panel for Brazilian Fanconi anemia patients

Mutation	Effect	Location	Gene	Reference
c.987_990delTCAC	p.His330Alafs*4	ex11	*FANCA*	Levran et al. (1997)
c.1115_1118delTTGG	p.Val372Alafs*42	ex 13	*FANCA*	Apostolou et al. (1996)
c.2535_2536delCT	p.Cys846Glnfs*19	ex 27	*FANCA*	Levran et al. (1997)
c.2853‐19_2853‐1del19	p.?^a^	29i	*FANCA*	Levran et al. (1997)
c.3788_3790delTCT	p.Phe1263del	ex 38	*FANCA*	Levran et al. (1997)
c.1393C>T	p.Gln465*	ex 13	*FANCC*	Yates et al. (2006)
c.65G>A	p.Trp22*	ex 1	*FANCC*	Gibson et al. (1996)
c.456+4A>T	p.Gly116_Asn152del	4i	*FANCC*	Whitney et al. (1993)
c. 67delG	p.Asp23Ilefs*23	ex 1	*FANCC*	Strathdee et al. (1992)
c.1077‐2A>G	p.?^a^	8i	*FANCG*	Demuth et al. (2000)
c.1480+1G>C	p.?^a^	ex11	*FANCG*	Auerbach et al. (2003)

a An effect at the protein level is expected but the exact nature is difficult to predict.

**Table 2:** PCR and ARMS‐PCR assays for detection of Fanconi anemia mutations

Mutation	Gene	Method	Primers	Product (bp)
c.2853‐19_2853‐1del19^a^	*FANCA*	PCR	30F: 5′‐GTCCCGAGCCGCCAGTC‐3′	380/361
			30R: 5′‐AAG GCA GAC CCA CCC TA AG‐3′	
c.1115_1118delTTGG^b^	*FANCA*	ARMS	1115delF: 5′‐GCT GAG TGC AGA GGA GTT AGC‐3′	234
			1115NF: 5′‐GCT GAG TGC AGA GGA GTT AGT‐3′	
			13R: 5′‐GTG GGA AGG GCT TCA CTG AG‐3′	
c.2535_2536delCT^b^	*FANCA*	ARMS	2535‐2536delF: 5′‐CAG CAG CAA TTT CTT ACT CTA TG‐3′	282
			2535‐2536NF: 5′‐CAG CAG CAA TTT CTT ACT CTA TC‐3′	
			2535‐2536R: 5′‐CTG CCT AAG CAG ACA GCA G‐3′	
c.987_990delTCAC^b^	*FANCA*	ARMS	987‐990delF: 5′‐ATA CCC TGA CTC AGA TAC TCA CA‐3′	220
			987‐990NF: 5′‐ATA CCC TGA CTC AGA TAC TCA CTC AC‐3′	
			987‐990R: 5′‐GTA ACA ATC TCA GGC ATC TG‐3′	
Internal Control^b^	HBB	ARMS	HBB‐F: 5′‐AGT CAG GGC AGA GCC ATC TA‐3′	377
			HBB‐R: 5′‐GCC CAT AAC AGC ATC AGG AG‐3′	

Primers and PCR conditions are adapted from Levran et al. (1997).

a PCR mixture to detect c.2853‐19_2853‐1del19: 10 ng DNA, 80 mM Tris‐HCl (pH 9.0), 20 mM (NH4)_2_SO_4_, 20 mM NaCl, 2 mM MgCl_2_, 0.2 mM dNTP, 0.4 μm each primer, and 0.5 U Taq DNA polymerase in final volume of 15 μL. Cycling: at 95°C/5 min; 30 cycles at 94°C/30 sec, 61°C/30 sec, and 72°C/30 sec, and finally 5 min at 72°C.

b ARMS‐PCR mixture: 20 ng DNA, 20 mM Tris‐HCl (pH 8.3), 50 mM KCl, 1.1–1.5 mM MgCl_2_, 0.2 mM dNTP, 0.6 μm each primer, and 0.5 U Taq DNA polymerase in final volume of 15 μL. Cycling: 95°C/5 min; 30 cycles at 94°C/30 sec, annealing at 65°C/30 sec for c.1115_1118delTTGG, 61°C/30 sec for c.2535_2536delCT and 64°C/30 sec for c.987_990delTCAC followed by 72°C/30 sec with final cycle at 72°C/5 min. PCR, polymerase chain reaction, ARMS, amplification‐refractory mutation system.

**Table 3:** Restriction assays (PCR‐RFLP) for detection of Fanconi anemia mutations

Mutation	Gene	Primers	Restriction enzyme	Product (bp)
c.67delG^a^	*FANCC*	F: 5′‐ACC ATT TCC TTC AGT GCT GG‐3′	*Bsp*1286I	Wild type: 129, 22
		R: 5′‐GTT TCC AAA GTG GAA GCC TGA GCC‐3′		Mutated: 151
c.65G>A^a^	*FANCC*	F: 5′‐TGG ATG CAG AAG CTT TCT GGA T‐3′	*Fok*I	Wild type: 210, 15, 17
		R: 5′‐TCC ATC GGC ACT TCA GTC AA‐3′		Mutated: 227, 15
c.1393C>T^a^	*FANCC*	F: 5′‐AGA AGC AGC AGC CTC TCA GC‐3′	*BssK*I	Wild type: 21, 103, 19, 16, 28
		R: 5′‐ATG CTG GAC CAC AGG GAG AC‐3′		Mutated: 124, 19, 16, 28
c.1480+1G>C^a^	*FANCG*	F: 5′‐CAC ACC TGA GGA AAA AGA ACA A‐3′	*Hph*I	Wild type: 135, 146, 18
		R: 5′‐CAA GAA GTG TCT TCC CAG CC‐3′		Mutated: 181, 18
c.456+4 A>T^b^	*FANCC*	F: 5′‐CTC ATA TAC TTT CAG CAC TCA G‐3′	*Sca*I	Wild type: 108, 23
		R: 5′‐TTT CAA AAG TGA TAA ATA TTA AGT AC‐3′		Mutated: 131
c.1077‐2A>G^c^	*FANCG*	F: 5′‐TCC TCA GGG CCC ATG AAC ATC CAT‐3′	*Dde*I	Wild type: 191
		R: 5′‐TGG GCC CCC AGA CTG GAC AGA C‐3′		Mutated: 250
c.3788_3790delTCT^c^	*FANCA*	F: 5′‐AGG ATT TAT GGC CTA GAT GTA AAA‐3′	*Mbo*II	Wild type: 130, 30
		R: 5′‐GAC GAC AGC AGG CCC ATC AAG GAA AA‐3′		Mutated: 160

Adapted from Gibson et al. (1996), Levran et al. (1997), Auerbach et al. (2003) and Yates et al. (2006).

a PCR mixture: 10 ng DNA, 20 mm Tris‐HCl (pH 8.3), 50 mm KCl, 1.5 mm MgCl_2_, 0.2 mm dNTP, 0.6 μm each primer, and 0.5 U Taq DNA polymerase in final volume of 15 μL. Cycling conditions for c.67delG, c.65G>A, c.1393 C>T, and c.1480+1G>C: 95°C/5 min followed by 30 cycles at 94°C/30 sec, annealing at 61°C/30 sec, 60°C/30 sec, 65°C/30 sec, and 58°C/30 sec, respectively, extension at 72°C/40 sec, and final cycle at 72°C/5 min.

b PCR mixture for c.456+4 A>T:10 ng DNA, 80 mm Tris‐HCl (pH 9.0), 20 mm (NH4)_2_SO_4_, 20 mm NaCl, 2 mm MgCl_2_, 0.2 mm dNTP, 0.4 μm each primer, and 0.5 U Taq DNA polymerase in final volume of 15 μL. Cycling conditions: 95°C/5 min, 30 cycles at 94°C/1 min, 51°C/1 min, 72°C/1 min, and final cycle at 72°C/5 min.

c PCR for c.1077‐2A>G was according to Auerbach et al. (2003), and for c.3788_3790delTCT followed Levran et al. (1997). Restriction assays were performed according to manufactures instructions for each enzyme. PCR, polymerase chain reaction; RFLP, restriction fragment length polymorphism.

The PCR and ARMS‐PCR methods and also the primers for c.1115_1118delTTGG and c.2853‐19_2853‐1del19 were adapted from Levran et al. (1997). Primers for the mutations c.2535_2536delCT and c.987_990delTCAC were designed using Primer3Plus software (Untergasser et al. 2007). All PCR and ARMS‐PCR primers are listed in Table 2. The restriction assays and also primers for c.67delG; c.65G>A and c.456+4A>T were adapted from the original publications (Gibson et al. 1996; Auerbach et al. 2003; Yates et al. 2006). Primers and suitable enzymes for mutations c.1393C>T and c.1480+1G>C were selected using PCR designer program (http://primer1.soton.ac.uk) (Ke et al. 2002). The mutation c.3788_3790delTCT was detected by mismatch PCR assay followed by a restriction assay developed by Levran et al. (1997) and detection of c.1077‐2A>G was according to Auerbach et al. (2003). All PCR‐RFLP primers are listed in Table 3.

### Detection of *FANCA* large deletion by MLPA

MLPA was used to detect deletions and duplications of entire exons in the *FANCA* gene (Schouten et al. 2002). The Salsa MLPA kit with the probe mix P031 and P032 for *FANCA* (MRC Holland, Amsterdam, the Netherlands) was used according to the manufacturer's instructions (www.mlpa.com). Separation and quantification of MLPA products were done on ABI 3730 Genetic Analyzer (Applied Biosystems, Foster City, CA, USA). MLPA data were analyzed using GeneScan™ 500 TAMRA™ size standard (Applied Biosystems) and GeneMarker software (SoftGenetics, State College, PA, USA) as described in Ameziane et al. (2008).

### Sanger sequencing of *FANCA*,* FANCC*, and *FANCG*

In patients for whom only one mutation was identified by the screening test, the DNA sequence of the respective gene was further investigated using Sanger sequencing, except those with large deletions detected by MLPA. Sanger sequencing was performed with Big Dye Terminator (Applied Biosystems) followed by electrophoresis on ABI 3730 Genetic Analyzer. Sequence Pilot software (JSI Medical Systems, Kippenheim, Germany) and reference sequences from the RefSeq database (*FANCA* NM_000135.2; *FANCC* NM_000136.2; *FANCG* NM_004629.1) were used for data analysis. The reaction mixtures for the 25 μL PCR reactions were prepared as follows: 0.5 U Platinum Taq polymerase (Invitrogen, Carlsbad, CA, USA), 1.5 mm MgCl_2_, 0.2 mm dNTPs (Invitrogen), and 10 pmol primers. For the majority of amplicons, standard PCR conditions were used (initial denaturation at 95°C for 5 min, followed by 33 cycles of denaturation at 95°C for 30 sec, annealing at 60°C for 30 sec, and elongation at 72°C for 1 min). Some fragments required special conditions for PCR amplification including exons 5, 7, 13, 21, 26, 31, 38 of *FANCA* and exon 7 of *FANCC* with an annealing temperature at 55°C, and exon 1 of *FANCA* with annealing at 64°C and PCR mix supplementation with 10% DMSO. The procedure for sequencing FA genes and the primer sequences are described in Gille et al. (2012).

The pathogenic state of new mutations was investigated utilizing the in silico prediction algorithms SIFT, POLYPHEN2, and Align GVGD (Tavtigian et al. 2008; Kumar et al. 2009; Adzhubei et al. 2010), which are integrated in the Alamut software (Interactive Biosoftware, Rouen, France). All already known and novel mutations identified in this study were reported to the Fanconi Anemia Mutation Database (http://www.rockefeller.edu/fanconi), hosted by the Leiden University Medical Center, the Netherlands, Leiden Open Source Variation Database (LOVD v.3.0).

## Results

### Mutations identified by the screening panel

Mutations were identified in 128 of the 255 patients of the initial cohort. Twenty‐nine out of 128 patients were previously investigated. Twenty out of these 29 patients had the *FANCA* mutation c.3788_3790delTCT identified in an earlier study by our group (Magdalena et al. 2005). This mutation was homozygous in 12/20 and heterozygous in 8/20 patients, the latter being further investigated in this study for other alterations in *FANCA* to complete their genetic subtype. Nine out of 29 of these Brazilian patients (four homozygous and five compound heterozygous) harbored mutations which had been previously identified by Ameziane et al. (2008). The remaining 127/255 patients did not have any of their mutations detected in the screening test.

Overall, the screening test allowed the identification of at least one mutation in 50.2% (128/255) of the FA patients, 89 being in *FANCA*, 11 in *FANCC*, and 28 in *FANCG*. Homozygous mutations were found in 71 (55.5%) patients (43 in *FANCA*, 7 in *FANCC*, 21 in *FANCG*) and compound heterozygous in 57 (44.5%) patients (46 in *FANCA*, 4 in *FANCC*, 7 in *FANCG*). Only one mutation was identified in 53/57 patients, and 4/57 were compound heterozygous with both *FANCA* mutations detected by the screening test (Table 4).

**Table 4:** Fanconi anemia mutations identified by the screening panel

Mutation	Patients (128/255)	Homozygous: Heterozygous 71:57	Alleles (203/510)
	*FANCA* ‐ 89/255		136/510
c.3788_3790delTCT	69 (27.1%)	34:35	103 (20.2%)
c.2535_2536delCT	6 (2.4%)	3:3	11 (2.1%)^a^
c.987_990delTCAC	6 (2.4%)	2:4	9 (1.8%)^a^
c.2853‐19_2853‐1del19	5 (2.0%)	3:2	9 (1.8%)^a^
c.1115_1118delTTGG	3 (1.2%)	1:2	4 (0.8%)
	*FANCC* ‐ 11/255		18/510
c.65G>A	4 (1.6%)	4:0	8 (1.6%)
c.456+4 A>T	4 (1.6%)	2:2	6 (1.2%)
c.1393 C>T	3 (1.2%)	1:2	4 (0.8%)
	*FANCG* ‐ 28/255		49/510
c.1077‐2A>G	28 (11%)	21:7	49 (9.6%)

a The fact that mutation c.2535_2536delCT was detected in compound heterozygosis in two patients, and mutations c.987_990delTCAC and c.2853‐19_2853‐1del19 were simultaneously present in one patient explains the discrepancy between the number of alleles and the number of patients. Mutations c.67delG (*FANCC*) and c.1480+1G>C (*FANCG*) were not identified among the 255 patients with the methods used in the screening panel.

### Investigation of the second mutation using MLPA and Sanger sequencing

**Figure 1:**
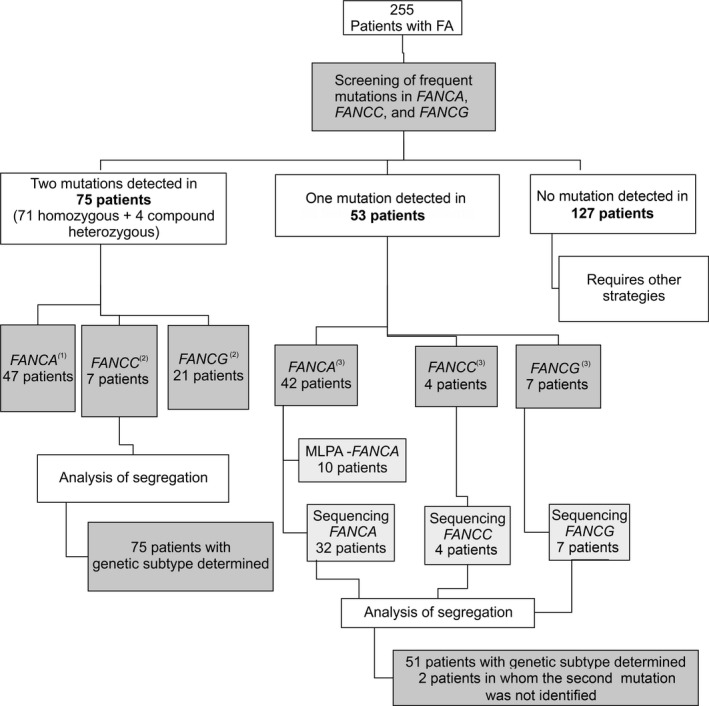
Strategy for the molecular investigation of patients with Fanconi anemia (FA). Homozygous or compound heterozygous: both mutations detected by the initial screening panel. Heterozygous: only one mutation detected by the initial screening panel and the second mutation investigated by multiplex ligation‐dependent probe amplification (MLPA) and/or Sanger sequencing. ^(1)^43 homozygous and 04 compound heterozygous patients. ^(2)^All homozygous patients. ^(3)^All heterozygous patients.

Using MLPA and Sanger sequencing, the second mutation was successfully identified in 51/53 heterozygous patients (40 in *FANCA*, 4 in *FANCC*, 7 in *FANCG*). And in two out of 42 *FANCA* heterozygous patients, the second mutation was not identified. Thus, a total of 126 patients had their mutations identified using the proposed strategy (Fig. 1).

Among the 40 *FANCA* heterozygous patients, 10 had large deletions detected by MLPA. Three of these patients had a large deletion encompassing exons 18–23 [c.(1626+1_1627‐1)_(2151+1_2152‐1)del]. And two large deletions [c.(1359+1_1360‐1)_(2778+1_2779‐1)del and c.(2778+1_2779‐1)_(2981+1_2982‐1)del] that had not been described yet were found.

The other 30/40 patients had the second *FANCA* mutation identified by Sanger sequencing. Twenty‐four different mutations were identified, of which 13 were novel (Table 5). Two of these novel variants, c.3163C>G (p.Arg1055Gly) and c.4199G>C (p.Arg1400Pro), were identified by affecting the same amino acids from those previously described disease‐causing mutation in the FA Mutation Database [c.3163C>T (p.Arg1055Trp) and c.4199G>A (p.Arg1400His)]. Two patients showed the splice site mutation c.190‐2A>T that is described in the Exome Aggregation Consortium (ExAC, http://exac.broadinstitute.org/) at an extremely low allele frequency (1.653e‐05). In silico analysis demonstrated that this variant results in aberrant splicing; and was thus considered as a new deleterious mutation. Among the *FANCA* variants identified by sequencing, the most frequent were c.718C>T detected in three patients, and c.190‐2A>T, c.3163C>G, c.3696delT, and c.4082A>C found in two patients each.

**Table 5:** *FANCA*,* FANCC*,* FANCG* mutations detected in the cohort of 128 FA patients

Exon	Mutation	Effect	Number of alleles	Reference
*FANCA*				
ex2	c.97delG	p.Glu33Lysfs*11	1	New mutation^a^
2i	c.190‐2A>T	p.?^b^	2	New mutation^a^
ex4	c.396dupT	p.His133Serfs*48	1	New mutation^a^
ex8	c.718C>T	p.Gln240*	3	Savoia et al. (1996)
ex8	c.784delA	p.Met262Cysfs*13	1	New mutation^a^
ex10	c.862G>T	p.Glu288*	1	FA Database^c^
ex11	c.987_990delTCAC	p.His330Alafs*4	9	Levran et al. (1997)
ex13	c.1115_1118delTTGG	p.Val372Alafs*42	4	Apostolou et al. (1996)
ex16	c.1508dupA	p.Tyr503*	1	New mutation^a^
ex27	c.2535_2536delCT	p.Cys846Glnfs*19	11	Levran et al. (1997)
ex28	c.2604_2609delTCAGTT	p.Gln869_Phe870del	1	Levran et al. (2005)
ex28	c.2636C>T	p.Arg879*	1	Ameziane et al. (2008)
28i	c.2778+1G>T	p.?^b^	1	FA Database^c^
ex29	c.2808G>C	p.Glu936Asp	1	New mutation^a^
ex29	c.2851C>T	p.Arg951Trp	1	Levran et al. (2005)
29i	c.2853‐19_2853‐1del19	p.?^b^	9	Levran et al. (1997)
ex32	c.3163C>G	p.Arg1055Gly	2	New mutation^a^
ex32	c.3166_3185dup	p.Trp1063Serfs*4	1	New mutation^a^
ex32	c.3239G>A	p.Arg1080Gln	1	Chandra et al. (2005)
ex36	c.3560dupG	p.His1188Thrfs*27	1	New mutation^a^
ex37	c.3638_3639delCT	p.Pro1213Argfs*64	1	Yagasaki et al. (2004)
ex37	c.3696delT	p.Phe1232Leufs*15	2	FA Database^c^
ex38	c.3788_3790delTCT	p.Phe1263del	103^d^	Levran et al. (1997)
ex40	c.4006T>G	p.Tyr1336Asp	1	New mutation^a^
40i	c.4010+2T>C	p.?^b^	1	New mutation^a^
ex41	c.4082A>C	p.Tyr1361Ser	2	New mutation^a^
ex41	c.4124_4125delCA	p.Thr1375Serfs*49	1	Savoia et al. (1996)
ex42	c.4198C>T	p.Arg1400Cys	1	Savoia et al. (1996)
ex42	c.4199G>C	p.Arg1400Pro	1	New mutation^a^
_1_36i	c.(?_‐42)_(3626+1_3627‐1)del^e^	p.0?^f^	1	FA Database^c^
3i_5i	c.(283+1_284‐1)_ (522+1_523‐1)del	p.?^b^	1	Ameziane et al. (2008)
5i_8i	c.(522+1_523‐1)_(792+1_793‐1)del	p.?^b^	1	Ameziane et al. (2008)
14i_28i	c.(1359+1_1360‐1)_(2778+1_2779‐1)del	p.?^b^	1	New mutation^a^
17i_23i	c.(1626+1_1627‐1)_(2151+1_2152‐1)del	p.?^b^	3	Ameziane et al. (2008)
17i_28i	c.(1626+1_1627‐1)_(2778+1_2779‐1)del	p.?^b^	1	FA Database^c^
21i_28i	c.(1900+1_1901‐1)_(2778+1_2779‐1)del	p.?^b^	1	Lo Ten Foe et al. (1996)
28i_30i	c.(2778+1_2779‐1)_(2981+1_2982‐1)del	p.?^b^	1	New mutation^a^
*FANCC*				
ex1	c.65G>A	p.Trp22*	8	Gibson et al. (1996)
ex4	c.338G>A	p.Trp113*	1	New mutation^a^
ex5	c.388delGinsAAAA	p.Glu130delinsLysLys	1	New mutation^a^
ex4	c.450_451insA	p.Asn152Lysfs	1	Ameziane et al. (2008)
4i	c.456+4 A>T	p.Gly116_Asn152del	6	Whitney et al. (1993)
ex9	c.996G>C	p.?^b^	1	Ameziane et al. (2008)
ex13	c.1393C>T	p.Gln465*	4	Yates et al. (2006)
*FANCG*				
ex1	c.60T>A	p.Asn20Lys	1	New mutation^a^
1i	c.84+3A>C	splice	1	New mutation^a^
ex03	c.256C>T	p.Gln86*	1	New mutation^a^
3i	c.307+1G>C	p.?^b^	1	Auerbach et al. (2003)
8i	c.1077‐2A>G	p.?^b^	49	Demuth et al. (2000)
ex10	c.1158delC	p.Ser387Profs*16	1	FA Database^c^
ex10	c.1216dupC	p.Gln406Profs*13	1	New mutation^a^
10i	c.1433+1G>A	p.?^b^	1	New mutation^a^
TOTAL	52 mutations		254 alleles	22 New mutations

Nomenclature and variant descriptions followed the Human Genome Variation Society instructions (http://www.HGVS.org/varnomen) and the cDNA reference sequences are from the RefSeq database (*FANCA*: NM_000135.2; *FANCC*: NM_000136.2; *FANCG*: NM_004629.1). The nucleotide numbering uses the A of the ATG translation initiation start site as nucleotide +1.

a The novel mutations identified in this study were recently included in the FA Mutation Database.

b An effect at the protein level is expected but the exact nature is difficult to predict.

c Available at: http://www.rockefeller.edu/fanconi. FA, Fanconi anemia.

d In 2/128 patients, only c.3788_3790delTCT mutation was detected, which explains that only 254 out of 256 potential alleles were identified.

e Deletion starting somewhere upstream from the 5' end of the gene, located at coding DNA nucleotide ‐ 42.

f Probably no protein is produced.

In all four *FANCC* heterozygotes, the second pathogenic mutation was identified by Sanger sequencing, and two of them were new variants (c.338G>A, c.388delGinsAAAA).

Seven different mutations were found among the seven *FANCG* heterozygous patients, five are novel and three of seven are located in exon 10 (Table 5).

Overall, a total of 52 mutations were found in this cohort, 30 of which had already been described in the literature and 22 (42.3%) were novel mutations (Tables 5, 6a,b). Thus, screening for the 11 common mutations, and when necessary further testing with MLPA and Sanger sequencing, led to the molecular characterization of 126/255 Brazilian FA patients.

**Table 6:** Novel mutations detected in *FANCA, FANCC* and *FANCG* (a) New mutations with deleterious effects (b) New mutations with probable deleterious effects

Number of patients	Location	Mutation	Effect	Type of mutation/predicted consequence
(a)				
2^a^	*FANCA*/2i	c.190‐2A>T	p.?^b^	Substitution ‐ splicing affected^b^
1	*FANCA*/ex2	c.97delG	p.Glu33Lysfs*11	Deletion ‐ frameshift
1	*FANCA*/ex4	c.396dupT	p.His133Serfs*48	Duplication ‐ frameshift
1	*FANCA*/ex8	c.784delA	p.Met262Cysfs*13	Deletion ‐ frameshift
1	*FANCG*/10i	c.1433+1G>A	p.?^b^	Substitution ‐ splicing affected^b^
1	*FANCA*/ex16	c.1508dupA	p.Tyr503*	Duplication ‐ nonsense
1	*FANCA*/ex32	c.3166_3185dup	p.Trp1063Serfs*4	Duplication ‐ frameshift
1	*FANCA*/ex36	c.3560dupG	p.His1188Thrfs*27	Duplication ‐ frameshift
1	*FANCA/*40i	c.4010+2T>C	p.?^b^	Substitution ‐ splicing affected^b^
1	*FANCA*/14i_28i	c.(1359+1_1360‐1)_(2778+1_2779‐1)del	p.?^b^	Large deletion
1	*FANCA*/28i_30i	c.(2778+1_2779‐1)_(2981+1_2982‐1)del	p.?^b^	Large deletion
1	*FANCC*/ex4	c.338G>A	p.Trp113*	Substitution ‐ nonsense
1	*FANCG*/ex3	c.256C>T	p.Gln86*	Substitution ‐ nonsense
1	*FANCG*/ex10	c.1216dupC	p.Gln406Profs*13	Duplication ‐ frameshift

Number of patients	Location	Mutation	Effect	Type of mutation/predicted consequence			
					Align GVGD	SIFT	Polyphen2
(b)							
1	*FANCA*/ex29	c.2808G>C	p.Glu936Asp	Missense	C35	Deleterious, score: 0	Probably damaging score: 0.991
2^a^	*FANCA*/ex32	c.3163C>G	p.Arg1055Gly	Missense	C65	Deleterious, score: 0	Probably damaging score: 0.998
1	*FANCA*/ex40	c.4006T>G	p.Tyr1336Asp	Missense	C65	Deleterious, score: 0	Probably damaging score: 0.933
2^a^	*FANCA*/ex41	c.4082A>C	p.Tyr1361Ser	Missense	C65	Deleterious, score: 0	Probably damaging score: 0.997
1	*FANCA*/ex42	c.4199G>C	p.Arg1400Pro	Missense	C65	Deleterious, score: 0	Probably damaging score: 0.986
1	*FANCG*/ex1	c.60T>A	p.Asn20Lys	Missense	C0	Tolerated, score: 0.07	Probably damaging score: 0.994
1	*FANCC*/ex5	c.388delGinsAAAA	p.Glu130delinsLysLys	Deletion/insertion ‐ in frame			
1	*FANCG*/1i	c.84+3A>C	p.?^b^	Substitution ‐ probable splicing affected^c^			

All new mutations were identified heterozygously with another pathogenic mutation in the same gene.

a Mutation identified in two nonconsanguineous patients.

b An effect at the protein level is expected but the exact nature is difficult to predict.

c The effect of the splice site change on RNA was not investigated.

### Segregation of mutations in Fanconi anemia families

In 95 of 126 patients (75.4%), the segregation of at least one allele was confirmed by the analysis of paternal and/or maternal samples. Segregation analysis was not possible in 31 patients due to the unavailability of maternal and/or paternal samples.

### Novel mutations

The 22 novel mutations detected among the 126 patients are outlined on Table 6a,b. Fifteen correspond to changes in *FANCA* gene, two in *FANCC*, and five in *FANCG*. Of these, 14 were considered to be deleterious because of their functional consequences (frameshift, nonsense, large deletions and splicing affecting mutations +1; +2 and −2). With regard to the other eight mutations comprised of missense, in frame or splicing affecting mutations +3, the in silico analysis suggests that they are potentially pathogenic; however, confirmation of their effects on the normal function of the genes products is required.

### Costs and time estimate

The cost estimate per patient in each step of the proposed algorithm was US$ 60.15 for screening of common mutations, US$ 75.30 for MLPA of *FANCA*, US$ 643.15 for *FANCA* sequencing, and US$ 245.20 for *FANCC* as well as for *FANCG* sequencing. The estimated cost per patient using this strategy ranged from US$ 60.15 to US$ 1269.00 with an average cost of US$ 703.70. These estimates were based on the number of patients investigated in each phase of the strategy (Fig. 1) and the frequency of FA‐A, FA‐C, and FA‐G complementation groups in the literature. If the screening is not utilized and the search for FA mutations starts straight from MLPA and Sanger sequencing of *FANCA*, proceeding to *FANCC* and *FANCG* when needed, the estimated cost could range from US$ 718.50 to US$ 1209.00 with an average of US$ 905.00 considering the literature information about frequency of FA complementation groups.

The screening of common mutations requires 5 days, MLPA 3 days, sequencing of *FANCA* 12 days, and sequencing of *FANCC* and *FANCG* 3.5 days each; therefore the turnaround time per patient ranged from 5 to 23.5 days with an average of 11 days. This estimate considered the number of patients identified in each phase of the algorithm (Fig. 1) and the frequency of FA complementation groups in the literature. However, the turnaround time could range from 15 to 22 days with an average time of 13 days if the search for FA mutations starts by MLPA and *FANCA* sequencing without the initial screening for common mutations.

## Discussion

The molecular investigation of FA in Brazilian patients, starting with a panel of common mutations, was proposed to determine the number of patients that could have their mutations identified with this strategy in order to make FA molecular subtyping cost and time efficient in routine clinical diagnostics.

*FANCA*,* FANCC*, and *FANCG* were selected to compose the screening panel because they carry deleterious mutations in FA patients more frequently than do other FA genes. The frequency of genetic subtypes in different populations has shown that 85 to 90% of patients are included in the FA‐A, FA‐C, and FA‐G subtypes, whereas the remaining are distributed among the other 18 genetic subtypes, and a few cases are still awaiting for genetic classification (Levitus et al. 2006; Auerbach 2009; Ameziane et al. 2012; Gille et al. 2012; Schneider et al. 2015). This screening test, despite including only three genes and 11 mutations, allowed the identification of at least one FA mutation in 50.2% (128/255) of this cohort (Table 4). Considering the high genetic heterogeneity of FA, this strategy demonstrated its relevance to the molecular characterization of Brazilian FA patients. Such knowledge of the recurrent mutations in certain ethnic groups or geographical locations allows for dedicated screening approaches in the Brazilian population, which could also be applied to other populations.

Mutations c.3788_3790delTCT and c.1115_1118delTTGG included in the screening panel are the most frequent in *FANCA* (Levran et al. 1997, 2005; Castella et al. 2011). In this study, 69 out of 255 patients (27.1%) carried c.3788_3790delTCT mutation (Table 4), and it is in agreement with our previous report where this genetic alteration was found in 24/80 (30%) of Brazilian FA patients (Magdalena et al. 2005). In contrast, c.1115_1118delTTGG was detected in 1.2% of this cohort (Table 4), whereas Levran et al. (2005) had reported a frequency of 2.2% in Brazilian patients and 5.5% in other ethnic backgrounds. In a Spanish FA‐A subtype cohort, frequencies of 20.7% for c.3788_3790delTCT and 9.4% for c.1115_1118delTTGG were observed (Castella et al. 2011).

The c.456+4A>T *FANCC* mutation, highly frequent among Ashkenazi Jews, was detected in only four of 255 patients (1.6%), and c.67delG, common among Europeans, was not identified in this Brazilian cohort. These findings enhance the previous observations about the differences in prevalence of some mutations in certain ethnic groups (Faivre et al. 2000; Futaki et al. 2000; Kutler et al. 2003; Kutler and Auerbach 2004; Neveling et al. 2009). *FANCC* mutations c.65G>A and c.1393C>T were also identified at low frequencies, 1.6% and 1.2%, respectively (Table 4), but higher than those reported in the FA database. This higher frequency was expected for c.1393C>T since it was first identified in Brazilian patients by Yates et al.(2006). Finally, *FANCG* c.1077‐2A>G, initially described by Demuth et al. (2000), was found to be the second most frequent mutation in this study. It was detected in 28 of 255 (11%) patients (Table 4) supporting the previous data of Auerbach et al. (2003) where all seven FA‐G Brazilian patients presented this mutation.

The screening panel developed for this study allowed the identification of homozygous mutations in 71 out of the 255 patients and compound heterozygous mutations in 4/255 patients. Among 53/255 heterozygous patients, two were excluded because only one pathogenic mutation was detected, probably owing to somatic mosaicism that can lead to reversion from the mutant to the wild‐type allele (Waisfisz et al. 1999; Ameziane et al. 2008; Neveling et al. 2009; Castella et al. 2011; Gille et al. 2012). To identify the second mutation in the remaining 51 heterozygous patients (40 in *FANCA*, 4 in *FANCC*, and 7 in *FANCG*), additional analysis was required using MLPA and/or Sanger sequencing of the entire respective gene (Fig. 1).

Among the 40 *FANCA* compound heterozygous patients 25% had large deletions. Identification of this type of genetic alteration is essential for the molecular diagnosis of FA as 15–40% of pathogenic mutations in *FANCA* are caused by large deletions (Ameziane et al. 2012; Gille et al. 2012).

The strategy of starting with the screening for common mutations reduced the need for MLPA and (or) sequencing analysis in 29.4% of the cases, as 75/255 had the two mutations detected by the initial screening. Furthermore, this approach allowed the sequencing of a single gene in 20.0% of the heterozygous patients, as the second mutation is likely to be detected in the same gene previously implicated in the disorder.

The sequencing data of FA genes in those patients whose second mutation was not identified by the screening panel showed that some *FANCA* exons were more frequently mutated among the Brazilian patients. This information led to the proposal of sequencing stratification into two sets according to the observed mutation frequencies. The first set would include exons 2, 8, 28, 32, 37, and 41 and the second set exons 4, 10, 16, 29, 36, 40, and 42. In most cases, this strategy would avoid the need to sequence the other 30 *FANCA* exons.

In total, 52 deleterious mutations were found in 126 molecularly characterized patients, including 17 point mutations, 18 small insertions and deletions, nine changes in splicing sites, and eight large deletions (Table 5). Of these variants, 22 (42.3%) are novel mutations not previously described (Table 6a,b). All novel mutations were heterozygous with another previously identified deleterious mutation in the same gene, which suggests their pathogenicity. Nevertheless, this might be a consequence of the initial strategy where only the compound heterozygous patients were sequenced. It is also very likely that patients homozygous for one of the novel mutations would exist as well. Most novel mutations were identified in only one patient, but three of the *FANCA* variants (c.190‐2A>T, c.3163C>G, and c.4082A>C) were found in two nonconsanguineous patients.

No functional studies or cDNA analyses have been performed to confirm the pathogenicity of these newly identified genetic changes. However, their pathogenicity is very likely according to the in silico evaluation using predictive algorithms such as SIFT, POLYPHEN2, and Align GVGD (Tavtigian et al. 2008; Kumar et al. 2009; Adzhubei et al. 2010).

The segregation of the mutant alleles in the families is another finding suggesting a pathogenic role for these novel mutations. It was possible to investigate the segregation of maternal and paternal alleles for 12 of the 22 novel variants. The large deletions were not investigated in parental samples because of their evident functional consequences. Furthermore, the definition of an inherited mutation enables the investigation of heterozygous carriers as well as genetic counseling. All FA patients with identified mutations should be referred to the genetic counseling, along with their parents and siblings whose mutation status should be characterized regardless of the manifestation of congenital malformations or clinical symptoms.

Genetic subtyping in this study showed that 69.0% of the patients who had molecular investigation concluded belonged to FA‐A, 8.7% to FA‐C, and 22.2% to FA‐G. Comparatively, Gille et al. (2012) evaluated patients from 11 nationalities and found that 57% were FA‐A, 11% FA‐C, and 9% FA‐G, whereas only one family was identified in each of the other groups (FA‐E, FA‐F, and FA‐B). Their results were similar to those found by other groups (Faivre et al. 2000; Kutler et al. 2003; Auerbach 2009). In the present cohort of Brazilian patients, it was found that 22.2% belonged to FA‐G subtype compared to the approximately 10% reported in the literature. This higher frequency might be due to the composition of the screening panel that included the *FANCG* mutation c.1077‐2A>G, which may introduce a bias in this frequency as investigations were only pursued for patients showing 1 of the 11 mutations in the initial panel.

All patients who had none of their mutations identified by the screening panel will be further investigated in subsequent studies. More comprehensive strategies such as complete sequencing of the *FANCA*,* FANCC*, and *FANCG* as well as next‐generation sequencing which allows for the simultaneous investigation of all FA genes will be utilized (Ameziane et al. 2012; Knies et al. 2012; De Rocco et al. 2014). The accurate frequency of FA subtypes among Brazilian patients will be possible only after conclusion of the molecular characterization of all 255 patients included in the initial cohort of this study.

In general, patients with FA require special clinical management and molecular diagnosis is necessary to rule out other diseases with overlapping phenotypes. A well‐established FA diagnosis requires a positive result from the chromosomal breakage test as well as the detection of pathogenic mutations in FA genes (Ameziane et al. 2012; Gille et al. 2012). The screening for the most frequent mutations together with MLPA and Sanger sequencing of the *FANCA, FANCC,* and *FANCG* genes enables the genetic subtyping of 50% of Brazilian patients.

The proposed approach led to reductions of 22% in cost and 16% in turnaround time, when compared to the cost of identifying mutations utilizing only MLPA and Sanger sequencing, thus enhancing the feasibility of the molecular characterization of FA patients in a routine diagnostic setting. Next‐generation sequencing has been presented as an alternative for sequencing genes with lower cost, but these methods are not feasible for all clinical laboratories worldwide and are not always suitable for the analysis of small numbers of samples at a time. The assignment of Brazilian patients to their genetic FA subtypes and the identification of their respective mutations provided data that were still underexplored in Brazil.

The knowledge of the genetic profiles of the Brazilian patients with Fanconi anemia will enable future studies to investigate if there is any influence of the distinct FA genotypes on the clinical course of this rare disease.

## Conflict of Interest

The authors declare no conflicts of interest related to this study.
